# Circulation patterns of human seasonal Influenza A viruses in Chile before H1N1pdm09 pandemic

**DOI:** 10.1038/s41598-021-00795-5

**Published:** 2021-11-02

**Authors:** Juan Mena, Rodrigo Tapia, Claudio Verdugo, Luis Avendaño, Paulina Parra-Castro, Rafael A. Medina, Gonzalo Barriga, Víctor Neira

**Affiliations:** 1 Programa de Doctorado en Ciencias Silvoagropecuarias y Veterinarias, Universidad de Chile, Santiago, Chile; 2 Departamento de Medicina Preventiva Animal, Facultad de Ciencias Veterinarias y Pecuarias, Universidad de Chile, Santiago, Chile; 3 Ecology and Evolution of Infectious Diseases Lab, Instituto de Patología Animal, Facultad de Ciencias Veterinarias, Universidad Austral de Chile, Valdivia, Chile; 4 Program of Virology, Faculty of Medicine, University of Chile, Santiago, Chile; 5 Departamento de Enfermedades Infecciosas e Inmunología Pediátrica, Escuela de Medicina, Pontificia Universidad Católica de Chile, Santiago, Chile; 6 Department of Microbiology, Icahn School of Medicine at Mount Sinai, Mount Sinai, NY 10029 USA; 7 Laboratory of Emerging Viruses, Virology Program, Institute of Biomedical Sciences, Faculty of Medicine, Universidad de Chile, Santiago, Chile

**Keywords:** Influenza virus, Viral infection

## Abstract

Understanding the diversity and circulation dynamics of seasonal influenza viruses is key to public health decision-making. The limited genetic information of pre-pandemic seasonal IAVs in Chile has made it difficult to accurately reconstruct the phylogenetic relationships of these viruses within the country. The objective of this study was to determine the genetic diversity of pre-pandemic human seasonal IAVs in Chile. We sequenced the complete genome of 42 historic IAV obtained between 1996 and 2007. The phylogeny was determined using HA sequences and complemented using other segments. Time-scale phylogenetic analyses revealed that the diversity of pre-pandemic human seasonal IAVs in Chile was influenced by continuous introductions of new A/H1N1 and A/H3N2 lineages and constant viral exchange between Chile and other countries every year. These results provide important knowledge about genetic diversity and evolutionary patterns of pre-pandemic human seasonal IAVs in Chile, which can help design optimal surveillance systems and prevention strategies. However, future studies with current sequences should be conducted.

## Introduction

Influenza A virus (IAV) is an important concern in public health, causing respiratory disease epidemics and between 290,000 and 650,000 deaths worldwide annually^1^. The last influenza pandemic was caused by a novel lineage of Influenza A/H1N1 (A/H1N1pdm09), causing more than 123,000 global deaths from March to December 2009^2^. This strain displaced the previous human seasonal IAV A/H1N1 subtype that was circulating before the pandemic^3^. Today, A/H1N1pdm09 is co-circulating seasonally with A/H3N2 and influenza B viruses^3^, and its viral dynamic is well known due to surveillance efforts and novel sequencing platforms^4,5^. Before the A/H1N1pdm09 pandemic, information about IAV genetic diversity was scarce, and only the HA gene was commonly sequenced. The viral dynamic of IAV circulating before the 2009 pandemic is still unknown in most of the world, especially in developing and least-developed countries.

The viral circulation is important to maintain seasonal IAV strains. Seasonal IAV is driven by introducing new lineages from other countries rather than a local persistence of lineages circulating from previous epidemics^6–9^. For example, studies suggest that A/H3N2 viruses originate from an ecological source located in East and Southeast Asia, and from there, spread to other regions of the world^9,10^. On the contrary, Asian regions play a limited role in disseminating new lineages of A/H1N1 viruses^3,10^. Also, a recently published complex metapopulation model of the spatial spread proposes several geographic areas act as potential sources of new variants^3,6,7^. In this way, the annual seasonal pattern is characterized by an increase in activity during the winter season in temperate regions^11^, and during the rainy season in the tropics^12^.

In South America and in general in the southern hemisphere, the epidemiological and evolutionary dynamics of circulating IAVs have only been partially explored due to the lack of IAV sequences. Some studies indicate that in South America IAV strains do not persist locally between seasons, and genetic diversity is driven by the northern regions of the continent, mainly influenced by North America^13–15^. In Chile, the information about IAV before the A/H1N1pdm09 pandemic is scarce. Only 43 HA sequences are publicly available, and there are no studies on the epidemiology and/or evolutionary dynamics of human IAVs. Therefore, this study aimed to determine the diversity of pre-pandemic human seasonal IAVs in Chile, filling the information gap in the region.

## Results

### Genetic evolution of pre-pandemic human seasonal IAVs in Chile

Human IAV isolates were genetically characterized to evaluate the diversity and genetic evolution of pre-pandemic human seasonal IAVs in Chile. Forty-two out of 57 IAV isolates obtained in this study were successfully whole-genome sequenced (GenBank accession numbers MN054079-MN055475). Those viruses were classified as subtypes H1N1 and H3N2. H1N1 viruses were isolated in 1996 (1) and 2000 (11), while H3N2 viruses were obtained in 1996 (11), 2001 (8), 2003 (2), 2004 (5), 2005 (3), and 2007 (1). In public depositories we found two IAV complete genomes, 30 HA, 17 NA, and 13M segments published before 2009, derived from H1N1, H1N2 and H3N2 subtypes. Fifty-four pre-pandemic IAV Chilean strains were classified as H3N2, 30 as H1N1, and 1 H1N2 subtypes. All Chilean pre-pandemic influenza sequences were incorporated in a table summarizing overall results (Table 1, Supplementary Table 1).

**Table 1:** Overall results of Chilean human-origin IAV obtained from 54 H3N2, 30 H1N1 and 1 H1N2 collected between 1994 and 2008.

	H1	H3	N1	N2	PB2	PB1	PA	NP	M	NS
1994	0	1	0	0	0	0	0	0	0	0
1996	1	12	1	11	1	1	1	1	1	1
1997	0	0	0	1	0	0	0	0	0	0
2000	12	3	12	0	12	12	12	12	12	12
2001	1	11	1	8	9	9	9	9	10	9
2002	1	0	0	0	0	0	0	0	0	0
2003	1	10	0	2	2	2	2	2	2	2
2004	0	5	0	5	5	5	5	5	5	5
2005	0	5	0	3	3	3	3	3	3	3
2006	3	3	0	2	0	0	0	0	1	0
2007	1	4	1	3	1	1	1	1	5	1
2008	11	0	11	0	0	0	0	0	7	0
Total	31	54	26	35	33	33	33	33	46	33

**Figure 1:**
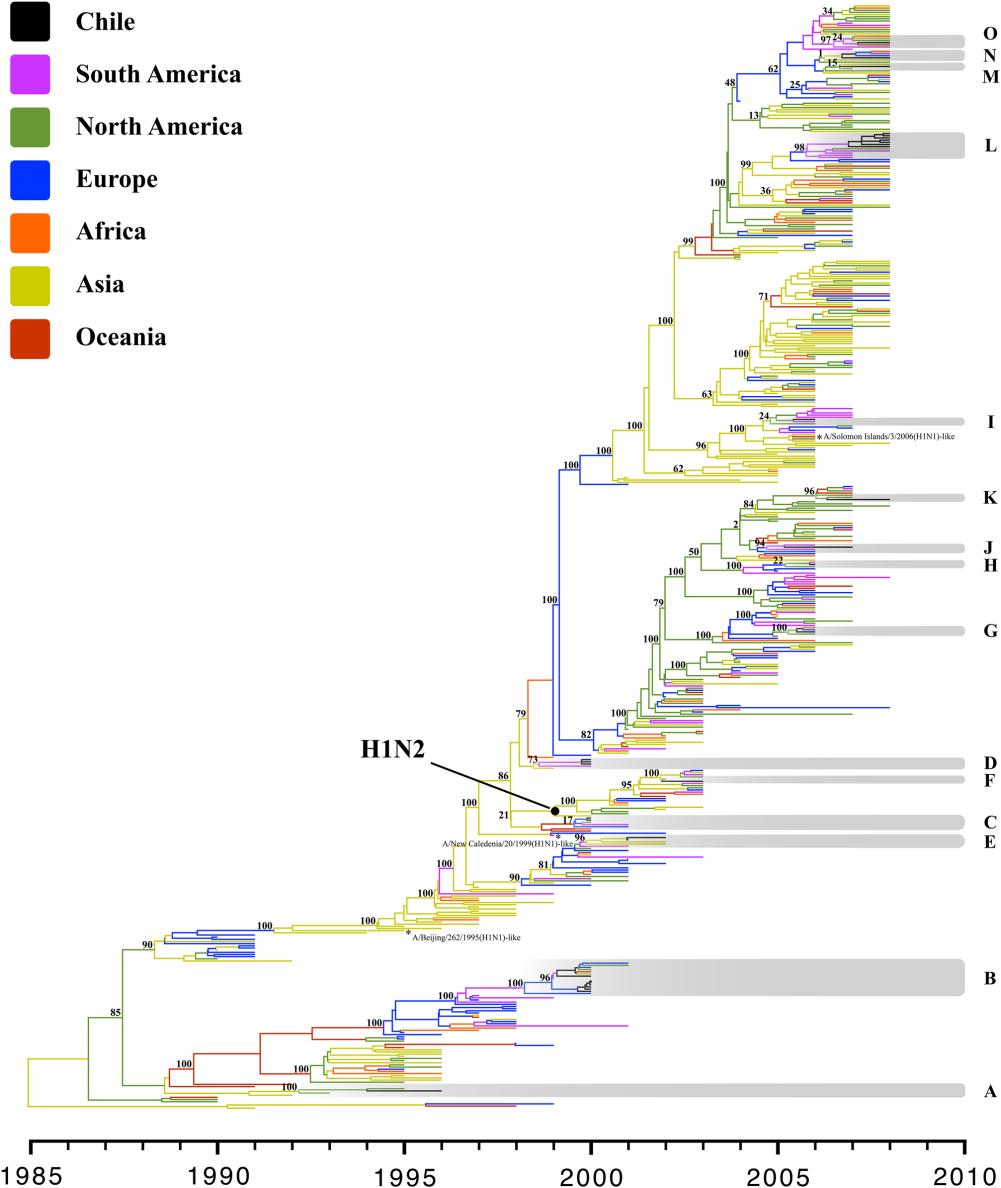
Time-scale Bayesian MCC tree of the HA1 portion of IAVs subtype H1 isolated around the world in the period 1990–2008. Branches are shaded by continent of origin. The genetic lineages that Chilean sequences group are highlighted and identified with the letters A-O. The H1N1 human seasonal influenza vaccine strains used in the southern hemisphere between 1999 and 2008 are included. The A/H1N2 genetic lineage is identified. The posterior probabilities are included for key nodes (Supplementary Table 2).

Time-scale phylogenetic analyses of the HA1 region were performed to study the H1 and H3 subtypes independently. The phylogeny showed that the H1 Chilean sequences are distributed in 15 different genetic lineages. According to node support (≥ 75% posterior probability), these sequences are related to viruses from different locations, especially from South and North America, and a lesser extent, from Asia (Fig. 1, Supplementary Table 2), evidencing an extensive global exchange of viruses between different geographic regions (continents). At least three lineages were observed co-circulating in Chile during the same year, especially in 2000, 2006, and 2008 (Fig. 1, Supplementary Table 2). The evolutionary analysis shows that the Chilean HA sequences are the last to appear in their respective genetic lineages, suggesting that Chile is one of the regions with the latest IAV arrival. An inter-seasonal extinction of Chilean H1 lineages was observed, as it is also observed in other geographical regions; however, some viruses were transmitted to other countries after they arrived in Chile in 2000, 2006 and 2008 (genetic lineages B, G, and N) (Fig. 1). The genetic lineages B and G are well supported with 100% posterior probability, but genetic lineage N with only 1% posterior probability. On the other hand, the H1N2 isolate (genetic lineage F) was grouped with viruses of the same subtype that were circulating globally between 2001 and 2003. The mean evolutionary rate for H1 subtype was 3.3 × 10^–3^ substitutions/site/year (95% highest probability density (HPD): 2.9–3.7 × 10^–3^ substitutions/site/year).

**Figure 2:**
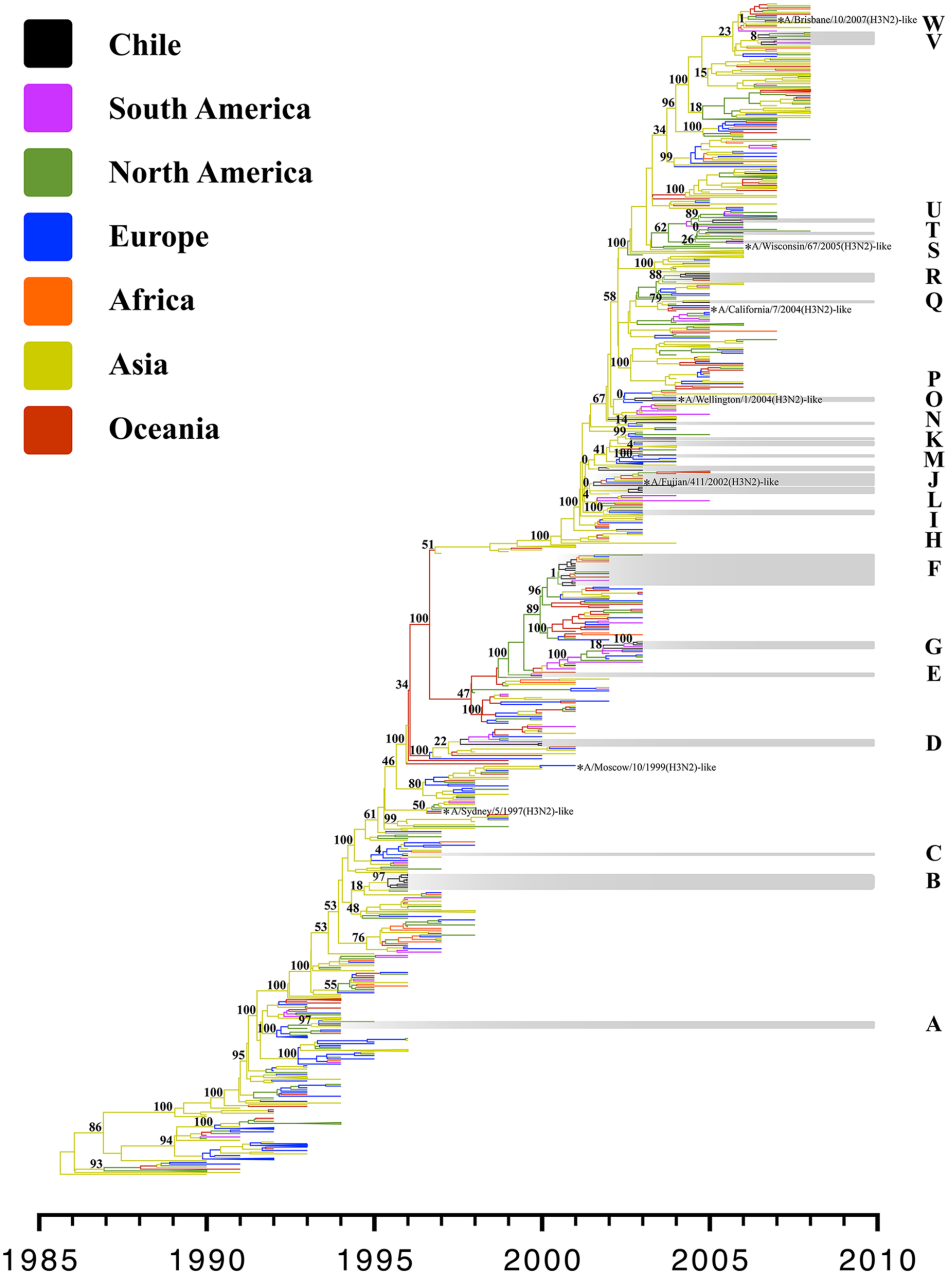
Time-scale Bayesian MCC tree of the HA1 portion of IAVs subtype H3 isolated around the world in the period 1990–2008. Branches are shaded by continent of origin. The genetic lineages that Chilean sequences group are highlighted and identified with the letters A-W. The H3N2 human seasonal influenza vaccine strains used in the southern hemisphere between 1999 and 2008 are included. The posterior probabilities are included for key nodes (Supplementary Table 2).

According to the time-scale phylogenetic analysis of the H3 subtype (Fig. 2), the mean evolutionary rate for the HA1 region was 3.9 × 10^–3^ substitutions/site/year (HPD 95%: 3.5–4.2 × 10^–3^ substitutions/site/year). As in the results obtained for the H1 subtype, an extensive global exchange of viruses between different geographic regions was identified. An inter-seasonal extinction of the Chilean H3 lineages was also evidenced. The phylogeny revealed that Chilean H3 sequences are distributed in 23 different genetic lineages (A–W) related to viruses from different geographic regions. Unlike the H1 subtype and based on well-supported genetic lineages (≥ 75% posterior probability), these sequences are commonly related to sequences from North America and Asia, and a lesser extent, from South America and Europe (Fig. 2, Supplementary Table 2). A co-circulation of two to five lineages in the same year was observed, specifically in 1996, 2000, 2003, 2004, 2005, 2006, and 2007. Viral interchange from Chile to other geographical regions was evidenced in at least eight opportunities, in 1994, 2000, 2001, 2003, 2004, 2006, and 2007 (genetic lineages A, D, F, G, J, L, U, and W) (Fig. 2); however, the only genetic lineages A or U are well supported with 97 and 89% posterior probability. The time to the most recent common ancestor (TMRCA) and related sequences (continent of origin) of Chilean H1 and H3 sequences are summarized in Supplementary Table 2.

The phylogenetic relationships of the rest of the genes NA, PB2, PB1, PA, NP, M, and NS were in concordance with the results observed for the HA trees (Supplementary Figs. 1, 2).

### Amino acid sequence analysis of human seasonal IAVs in Chile

**Figure 3:**
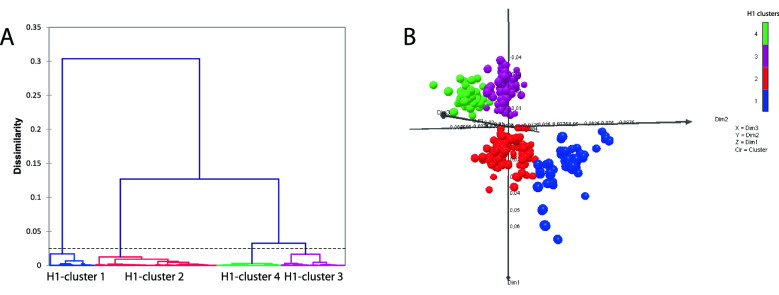
Genetic analysis of H1 influenza A viruses (IAVs) based on HA1 amino acid sequences. (**A**) Genetic clusters were defined by Ward’s method based on the Euclidean distances among the strains. (**B**) A 3-dimensional (3D) genetic map was constructed by Multidimensional Scaling (MDS) method. All axes represent amino acid distance (percent of distance) and the orientation of the map within these axes is free. Circles represent IAV strains used in this study. Color represents the genetic clusters: H1-cluster 1 is blue, H1-cluster 2 is red, H1-cluster 3 is purple, and H1-cluster 4 is green.

**Figure 4:**
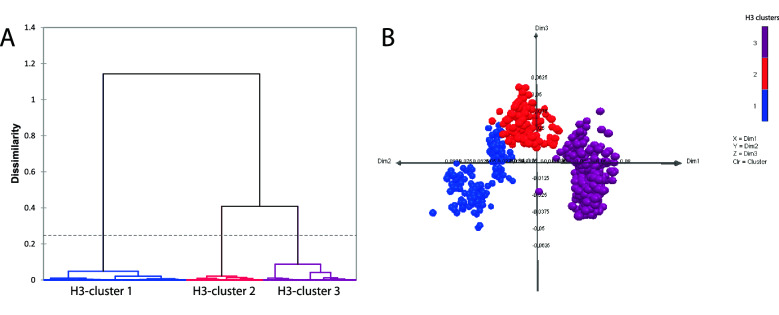
Genetic analysis of H3 influenza A viruses (IAVs) based on HA1 amino acid sequences. (**A**) Genetic clusters were defined by Ward’s method based on the Euclidean distances among the strains. (**B**) A 3-dimensional (3D) genetic map was constructed by Multidimensional Scaling (MDS) method. All axes represent amino acid distance (percent of distance) and the orientation of the map within these axes is free. Circles represent IAV strains used in this study. Color represents the genetic clusters: H3-cluster 1 is blue, H3-cluster 2 is red, and H3-cluster 3 is purple.

Genetic analyses based on HA1 amino acid sequences were performed to determine the genetic clusters and to predict potential antigenic evolution of worldwide human seasonal IAV sequences (1990–2008). These analyses show that these IAVs were grouped into four genetic clusters for H1 and three genetic clusters for H3 (Figs. 3, 4, respectively). For each subtype, clusters were named according to the year of emergence, with cluster 1 being the first to emerge. All clusters evidence circulation in all geographic regions, including South America and Chile. In general, Asia is the geographic region where IAV strains from each genetic cluster were isolated for the first time, while South America, including Chile, is the region where IAV strains from each genetic cluster were isolated for the last time. This could suggest that this clusters generally originate from Asia and finally reach South America. However, this cannot be confirmed due to limited data. Co-circulation of different IAV clusters of the same subtype in Chile was also observed in 2000, 2006, and 2008 for subtype H1, while for subtype H3 in 2003 (Supplementary Tables 2, 3).

## Discussion

We evaluated the genetic diversity and evolution of the seasonal human IAVs in Chile between 1994 and 2008. IAV subtypes A/H1N1, A/H1N2, and A/H3N2 co-circulated in the Chilean population before the 2009 pandemic. The A/H3N2 subtype was the most commonly detected and sequenced in that period. These results are consistent with the global circulation patterns described for seasonal IAVs^3,10,16^. In general, A/H3N2 has been the dominant subtype since it first emerged in 1968^17^, despite the re-emergence of the H1N1 subtype in 1977^18^. The subtype A/H1N2 identified in this study in 2003 correspond to a reassortant virus that circulated around the world between 2001 and 2003^19^.

The average evolution rate estimated for the HA1 region of the H3 subtype is likely higher (3.9 × 10–3; HPD 95%: 3.5–4.2 × 10^–3^ substitutions/site/year) than that estimated for the H1 subtype (3.3 × 10–3; HPD 95%: 2.9–3.7 × 10^–3^ substitutions/site/year), which is consistent with previous studies^3,10^. As expected, phylogenetic analyses for H1 and H3 subtypes showed an extensive viral exchange between Chile and the other regions of the world, evidencing a continuous genetic flow inside and outside Chile, beyond a closed evolutionary system in the country. Pre-pandemic Chilean IAVs are mainly related to sequences from South America, North America, and Asia. These findings are consistent with previous studies, which reported that viruses arriving in South America originate mainly from North America and that there is a continuous viral exchange between South American countries^8,14,15^. However, A/H3N2 virus introductions would also come from Europe, indicating that the epidemic outbreaks in Chile every year are influenced by viruses from different geographical regions, which may differ antigenically. These results are also supported by the records of arrival of foreign tourists to Chile obtained between 2008 and 2021 by the Chilean Undersecretariat of Tourism, where a total of 48 117 494 tourists came mainly from South American countries (78.3%), Europeans (10.6%), North Americans (6.6%) and Asians (1.5%)^20^.

In general, previous studies have described that A/H3N2 lineages do not persist locally between epidemics, while A/H1N1 lineages can persist for several seasons and show more complex global dynamics^3^. In this study, a circulation of multiple A/H1N1 and A/H3N2 lineages was evidenced during the same season, which came from different geographic regions and generally disappeared at the end of each outbreak in Chile. This result shows a wide genetic diversity in each flu season in Chile, which is produced by introducing new A/H1N1 and A/H3N2 lineages from other countries rather than the local persistence of lineages from the previous season.

All IAV genetic clusters (based on amino acid sequences) determined in this study have circulated worldwide, showing the global distribution of this virus. In general, Asia is the geographic region where IAV strains from each genetic cluster were isolated for the first time. A similar situation occurs in Oceania, Europe, and North America. While Africa and South America, including Chile, are the regions where IAV strains from each genetic cluster were isolated for the last time. However, few sequences from Africa and South America have been published compared to the rest of the continents, and therefore there could be information bias. Although the surveillance was improved after the IAV pandemic in 2009, it is still insufficient in some countries, such as Chile. Previously, it has been shown that Asia plays an important role in transmitting seasonal human IAVs, showing that most lineages ultimately originated from this geographic region^3,6,9,21,22^.

Both sparse and bias sampling of specific geographic areas limit the interpretation of transmission patterns, and very similar IAV gene sequences from the same or different locations do not necessarily imply direct linkage, therefore, not reflecting the exact migration pathways of the virus^23^. For that reason, it is very important to qualify some interpretations of our phylogenies, such as the origin of the Chilean sequences and the inter-seasonal extinction of the viruses in Chile. In the first case, our phylogenies have an overrepresentation of sequences from North America, Asia, and Europe, and a scarce amount from Africa, Oceania, and South America. On the other hand, oversampling of specific geographic areas can lead to these areas becoming "sinks", where the overrepresentation of a geographic area causes phylogenetic estimates that viruses emerge from that geographic area^23^. In the second case, we identified that there would be an inter-seasonal extinction of viruses in Chile; however, despite the addition of 42 new IAV genomes, the total number of sequences are still insufficient spatially and temporally to ensure that there would not be a closed evolutionary system in the country from 1 year to another or from a couple of seasons.

Notably, the clusters determined by the genetic analysis carried out in this study, based on amino acid sequences, were similar (years of circulation) to the antigenic clusters obtained by previously published antigenic analyses, based on hemagglutination inhibition assay, specifically for the H3 subtype^22,24^. This result suggests that this method is a good tool to predict IAV antigenic evolution. However, we did not differentiate between some previously described antigenic clusters^24^, because there are only a few amino acid substitutions in the HA1 domain between the strains representing these clusters. Only one amino acid substitution in the HA1 domain can cause a high antigenic impact^25^.

In conclusion, the results obtained in this study indicate that pre-pandemic human seasonal IAVs in Chile are influenced by continuous introductions of viral variants from other geographic regions and that there is a continuous viral exchange between different countries. Moreover, a wide genetic diversity was observed co-circulating in the same season in Chile. This is the first study on human IAV phylodynamic in Chile, providing important knowledge about genetic diversity and evolutionary patterns of human seasonal IAVs in Chile, which can help design optimal surveillance systems and prevention strategies. A limitation of this study was the small number of IAV sequences (data) published in South American countries, especially Chile. Greater IAV surveillance, sequencing and phylogeographic analyses are necessary to support these results, including post-pandemic IAVs that are currently circulating.

## Materials and methods

### Viruses

Fifty-seven human IAV pre-pandemic isolates were provided by the Virology Laboratory, Faculty of Medicine, University of Chile, Santiago, Chile. These isolates were collected in Santiago, Chile, from suspected patients, other epidemiological data is not available. The samples collection was performed using Vacuum-Assisted Nasopharyngeal Aspirates (NPAs) and the viral isolation attempted in Madin-Darby Canine Kidney (MDCK) cells, which were kindly provided by Dr. Goyal at University of Minnesota. The isolates were obtained from samples collected in 1996 (19 isolates), 2000 (11), 2001 (11), 2003 (3), 2004 (9), 2005 (3), and 2007 (1). The isolated were preserved at – 80 °C until sequencing. Informed consents were obtained from all subjects or their legal guardian(s). All methods and consent forms were performed in accordance with the relevant guidelines and regulations and approved by the Ethics Committee of the Faculty of Medicine at Universidad de Chile, and the Ethics Committee of the North Metropolitan Health Service, Chile (project Fondecyt Nº 194 0527 Apr 1994–Mar 1997; Nº 194 0527; and Fondecyt Nº 198 0892 Apr 1998 – Mar 2007).

### Virus confirmation and propagation

First the IAV isolates were tested using real time RT-PCR (rRT-PCR) to reconfirm the IAV presence. After, isolates were propagated in MDCK cells to yield enough virus to attempt the whole genome sequencing. Briefly, RNA was extracted by TRIzol LS Reagent (Invitrogen, Carlsbad, CA, USA) and tested by rRT-PCR, amplifying a conserved region of the matrix gene^26^. rRT-PCR positive isolates were propagated in MDCK cells previously cultured using minimum essential medium (MEM) supplemented with 10% fetal calf serum (FCS) and 1% antibiotic–antimycotic solution^27^. Confluent MDCK monolayers were washed twice with PBS containing 1 μg/mL of trypsin treated with *N*-tosyl-l-phenylalanyl chloromethyl ketone (TPCK) (Sigma-Aldrich, St. Louis, Mo, USA), inoculated with each IAV isolate, and incubated for virus absorption for 1 h at 37 °C. Subsequently, cells were rinsed with PBS to eliminate the unbound virus, and an IAV growth medium (MEM supplemented with 1 μg/mL of TPCK-treated trypsin, 0.3% bovine serum albumin, and 1% antibiotic–antimycotic solution) was added. The monolayers were incubated at 37 °C and observed for cytopathic effect (CPE) daily for 5 days. The supernatants of cultures without CPE were re-inoculated in MDCK cells and observed for another 5 days^28^. Isolates were tested by rRT-PCR and Hemagglutination assay to confirm the presence of IAV^28,29^. RNA was submitted to the Molecular Virology Laboratory, Pontificia Universidad Católica de Chile for further steps.

### Whole genome sequencing

The whole IAV genome was amplified by a multisegment RT-PCR (mRT-PCR)^30^ and sequenced by Illumina. The mRT-PCR was performed at Molecular Virology Laboratory, Pontificia Universidad Católica de Chile. Briefly, RNA was subjected to reverse transcription and PCR amplification with the SuperScript III high-fidelity RT-PCR kit (Invitrogen, Carlsbad, CA, USA), using the primers Opti1-F1 (5-GT TA CGC GCC AGC AAA AGC AGG), Opti1-F2 (5-GTT ACG CGC CAG CGA AAG CAG G), y Opti1-R1 (5′-GTT ACG CGC CAG TAG AAA CAA GG). A 50 μL total RT-PCR volume containing 25 μL of buffer, 0.35 μL of Opti1-F1, 0,65 of Opti1-F2 and 1 μL of Opti1-R1, 1 μL of Enzyme Mix, 17 μL of water, and 5 μL of RNA was performed. The thermal cycler program consisted of one cycle of 55 °C for 2 min, 42 °C for 60 min and 94 °C for 2 min, five cycles of 94 °C for 30 s, 44 °C for 30 s and 68 °C for 3,5 min; 26 cycles of 94 °C for 30 s, 57 °C for 30 s and 68 °C for 3.5 min, and one cycle of 68 °C for 10 min. PCR products were purified using Agencourt AMPure XP 5-ml kit (Beckman Coulter, Brea, CA, USA), and those with ≥ 25 ng/μL of DNA concentration were submitted for sequencing. The purified PCR products were submitted to the Center for Research on Influenza Pathogenesis (CRIP), Icahn School of Medicine at Mount Sinai (New York City, NY, USA) for sequencing on an Illumina HiSeq 2000 sequencer.

### Phylogenetic analysis

Phylogenetic analyzes for all IAV segments were performed. Sequence alignments were separately constructed for HA (H1 and H3 subtypes), NA (N1 and N2 subtypes) and each internal gene segment (PB2, PB1, PA, NP, M, and NS), using MUSCLE v3.8.3^31^. Reference sequences available in Global Initiative on Sharing All Influenza Data (GISAID) EpiFlu Database^32^ were incorporated, including all Chilean sequences available. The CD-HIT program^33^ was used to cluster the sequences according to each continent’s genetic diversity per year and thus select some representative sequences of each cluster. This allowed us to reduce the data sets for the construction of phylogenetic trees. The phylogenetic trees were constructed by the maximum likelihood method using IQ-TREE with substitution model selection (ModelFinder implememted in IQ-TREE) option and 1000 bootstraps^34^.

Additionally, for the HA segment, the encoded HA1 domain was analyzed using time-scaled Bayesian analyses. The HA1 domain is the most variable region of the virus^35–38^; therefore, it is selected for the time scaled tree^14^. The number of sequences by geographic origin in the H1 subtype database was as follows (number in parentheses indicate number of sequences): Chile (31), South America (53), North America (119), Europe (89), Africa (39), Asia (145) and Oceania (34); and for H3 subtype was as follow: Chile (50), South America (47), North America (147), Europe (141), Africa (32), Asia (238) and Oceania (80). Phylogenetic relationships of the HA from subtypes H1 (510) and H3 (735) were inferred for each data set separately using the time-scaled Bayesian approach using Markov chain Monte Carlo (MCMC) methods available via the BEAST v1.10.4 package^39^. Two clock models including strict clock and uncorrelated lognormal (UCLN) relaxed clocks, and four demographic models (constant size, exponential growth, logistic growth and expansion growth) were tested independently. The best molecular clock model was tested by marginal likelihood estimation (MLE)^40^. An UCLN molecular clock was used, with a general-time reversible (GTR) model of nucleotide substitution with a gamma-distributed rate variation among sites. For the H1 subtype, we used an expansion growth demographic model, while for the H3 subtype, we used a logistic population size model. The MCMC was run for at least 200 million iterations, with sub-sampling every 10,000 iterations for each data set. The BEAGLE library was used to improve computational performance^41^. All parameters reached convergence, as assessed visually using Tracer v1.6 (http://tree.bio.ed.ac.uk/software/tracer/), with statistical uncertainty reflected by values of the 95% highest posterior density (HDP). The initial 10% of the chain was removed as burn-in, and maximum clade credibility (MCC) trees were summarized using TreeAnnotator v1.8.0^42^. Chilean sequences that are grouped into genetic lineages that are supported by a ≥ 75% posterior probability will be considered for inferring the main results from the phylogenies.

### Amino acid sequence analysis

We analyzed amino acid sequences, deduced from nucleotide sequences, encoding the HA1 domain (327 amino acids for subtype H1 and 329 amino acids for subtype H3). The sequence alignment was done with MUSCLE v3.8.3^31^. We computed pairwise distances between amino acid sequences to construct a dissimilarity matrix, using the method p-distance in MEGA software (version 7.0.26)^43^. Genetic clusters were defined by Ward’s method based on the Euclidean distances among strains, and a 3-dimensional (3D) genetic map was constructed with Multidimensional Scaling (MDS) method, using the XLSTAT software (version 2020.1.2)^25^. Worldwide human seasonal IAV sequences, between 1990 and 2008, were included in the analyses. Reference sequences were obtained from GISAID EpiFlu Database^32^.

## Supplementary Information

Supplementary Information.
