# Supplementary material for: Circulation patterns of human seasonal Influenza A viruses in Chile before H1N1pdm09 pandemic
Source: Sci Rep. 2021 Nov 2;11:21469. doi: 10.1038/s41598-021-00795-5 (PMC8564531; doi:10.1038/s41598-021-00795-5)
Supplement: Supplementary file 1 — Supplementary Information. [file 41598_2021_795_MOESM1_ESM.docx]

**Supplementary material**

Circulation patterns of human seasonal Influenza A viruses in Chile before H1N1pdm09 pandemic.

Juan Mena^1,2^, Rodrigo Tapia^2^, Claudio Verdugo^3^, Luis Avendaño^4^, Paulina Parra-Castro^5^, Rafael Medina^5,6^, Gonzalo Barriga^7^, Victor Neira^2,^*

**Supplementary figure 1**. Phylogenetic analysis of the PB2, PB1, PA, NP, NA, M and NS genes of IAVs subtype H1. The phylogenetic trees were inferred using the maximum likelihood method in IQTREE based on GTR+F+I+G4 (PB2, PB1, PA, NA and M) and TVM+F+I+G4 (NP, NS) nucleotide substitution model with 1000 bootstrap replicates. Chilean and references sequences are represented in different colors.

**Supplementary figure 2**. Phylogenetic analysis of the PB2, PB1, PA, NP, NA, MP and NS genes of IAVs subtype H3. The phylogenetic trees were inferred using the maximum likelihood method in IQTREE based on GTR+F+I+G4 (PB2, PB1, PA and NA) and TVM+F+I+G4 (NP, M and NS) nucleotide substitution model with 1000 bootstrap replicates. Chilean and references sequences are represented in different colors.

**Supplementary Table 1**. Overall results of Chilean human-origin IAV sequences obtained between 1994 and 2008.

| Year isolation | H1N1 subtype | | | | | | | | | | | | | | | | | | | |
| --- | --- | --- | --- | --- | --- | --- | --- | --- | --- | --- | --- | --- | --- | --- | --- | --- | --- | --- | --- | --- |
|  | **Study** | | | | | | | | | |  | **Previously published** | | | | | | | | |
|  |  | **HA** | **NA** |  | **PB2** | **PB1** | **PA** | **NP** | **M** | **NS** |  | **HA** | **NA** |  | **PB2** | **PB1** | **PA** | **NP** | **M** | **NS** |
| 1996 |  | 1 | 1 |  | 1 | 1 | 1 | 1 | 1 | 1 |  | - | - |  | - | - | - | - | - | - |
| 2000 |  | 11 | 11 |  | 11 | 11 | 11 | 11 | 11 | 11 |  | 1 | 1 |  | 1 | 1 | 1 | 1 | 1 | 1 |
| 2001 |  | - | - |  | - | - | - | - | - | - |  | 1 | 1 |  | 1 | 1 | 1 | 1 | 1 | 1 |
| 2002 |  | - | - |  | - | - | - | - | - | - |  | 1 | - |  | - | - | - | - | - | - |
| 2006 |  | - | - |  | - | - | - | - | - | - |  | 3 | - |  | - | - | - | - | 1 | - |
| 2007 |  | - | - |  | - | - | - | - | - | - |  | 1 | 1 |  | - | - | - | - | - | - |
| 2008 |  | - | - |  | - | - | - | - | - | - |  | 11 | 11 |  | - | - | - | - | 7 | - |
| All |  | **12** | **12** |  | **12** | **12** | **12** | **12** | **12** | **12** |  | **18** | **14** |  | **2** | **2** | **2** | **2** | **10** | **2** |
|  |  |  |  |  |  |  |  |  |  |  |  |  |  |  |  |  |  |  |  |  |
| Year isolation |  | **H1N2 subtype** | | | | | | | | | | | | | | | | | | |
|  |  | **Study** | | | | | | | | |  | **Previously published** | | | | | | | | |
|  |  | **HA** | **NA** |  | **PB2** | **PB1** | **PA** | **NP** | **M** | **NS** |  | **HA** | **NA** |  | **PB2** | **PB1** | **PA** | **NP** | **M** | **NS** |
| 2003 |  | - | - |  | - | - | - | - | - | - |  | 1 | - |  | - | - | - | - | - | - |
| All |  | **0** | **0** |  | **0** | **0** | **0** | **0** | **0** | **0** |  | **1** | **0** |  | **0** | **0** | **0** | **0** | **0** | **0** |
|  |  |  |  |  |  |  |  |  |  |  |  |  |  |  |  |  |  |  |  |  |
| Year isolation | **H3N2 subtype** | | | | | | | | | | | | | | | | | | | |
|  | **Study** | | | | | | | | | |  | **Previously published** | | | | | | | | |
|  |  | **HA** | **NA** |  | **PB2** | **PB1** | **PA** | **NP** | **M** | **NS** |  | **HA** | **NA** |  | **PB2** | **PB1** | **PA** | **NP** | **M** | **NS** |
| 1994 |  | - | **-** |  | **-** | **-** | **-** | **-** | **-** | **-** |  | 1 | **-** |  | **-** | **-** | **-** | **-** | **-** | **-** |
| 1996 |  | 11 | 11 |  | 11 | 11 | 11 | 11 | 11 | 11 |  | 1 | **-** |  | **-** | **-** | **-** | **-** | **-** | **-** |
| 1997 |  | - | - |  | - | - | - | - | - | - |  | - | 1 |  | - | - | - | - | - | - |
| 2000 |  | - | - |  | - | - | - | - | - | - |  | 3 | - |  | - | - | - | - | - | - |
| 2001 |  | 8 | 8 |  | 8 | 8 | 8 | 8 | 8 | 8 |  | 3 | - |  | - | - | - | - | 1 | - |
| 2003 |  | 2 | 2 |  | 2 | 2 | 2 | 2 | 2 | 2 |  | 8 | - |  | - | - | - | - | - | - |
| 2004 |  | 5 | 5 |  | 5 | 5 | 5 | 5 | 5 | 5 |  | - | - |  | - | - | - | - | - | - |
| 2005 |  | 3 | 3 |  | 3 | 3 | 3 | 3 | 3 | 3 |  | 2 | - |  | - | - | - | - | - | - |
| 2006 |  | - | - |  | - | - | - | - | - | - |  | 3 | 2 |  | - | - | - | - | - | - |
| 2007 |  | 1 | 1 |  | 1 | 1 | 1 | 1 | 1 | 1 |  | 3 | 2 |  | - | - | - | - | 4 | - |
| All |  | **30** | 30 |  | **30** | **30** | **30** | **30** | **30** | **30** |  | **24** | **5** |  | **0** | **0** | **0** | **0** | **5** | **0** |

**Supplementary Table 2.** Clades, isolation year, time to the most recent common ancestor (TMRCA), related sequences (continent of origin) and clades posterior probabilities of Chilean HA sequences reported between 1994 and 2008.

| Clade |  | H1 subtype | | |  |  | Clade |  | H3 subtype | | |  |
| --- | --- | --- | --- | --- | --- | --- | --- | --- | --- | --- | --- | --- |
|  |  | **Year** | **Clade tMRCA** | **Continent** | **Posterior†** |  |  |  | **Year** | **Clade tMRCA** | **Continent** | **Posterior†** |
| A |  | 1996 | 1994 (1992-1994) | North America | 100 |  | **A*** |  | 1994 | 1993 (1993-1994) | Asia  Europe  North America | 97 |
| B* |  | 2000 | 1998 (1998-1999) | Europe  South America | 96 |  | **B** |  | 1996 | 1994 (1994-1995) | Asia | 14 |
| C |  | 2000 | 1999 (1998-1999) | Europe | 17 |  | **C** |  | 1996 | 1995 (1994-1995) | Europa  North America | 4 |
| D |  | 2000 | 1998 (1998-1998) | South America | 73 |  | **D*** |  | 2000 | 1997 (1996-1997) | Asia  South America | 22 |
| E |  | 2001  2002 | 1999 (1999-2000) | Asia | 96 |  | **E** |  | 2000 | 1998 (1997-1999) | North America  South America | 100 |
| F |  | 2003 | 2001 (2001-2002) | Asia  North America | 100 |  | **F*** |  | 2001 | 2000 (1999-2000) | North America  South America  Europe  Africa  Asia  Oceania | 1 |
| G* |  | 2006 | 2005 (2004-2005) | North America  Europe  Africa | 100 |  | **G*** |  | 2003 | 2001 (2001-2002) | South America North America | 18 |
| H |  | 2006 | 2005 (2004-2005) | North America South America | 22 |  | **H** |  | 2003 | 2001 (2001-2001) | Asia  Europe | 100 |
| I |  | 2006 | 2004 (2004-2005) | North America South America | 24 |  | **I** |  | 2003 | 2001 (2001-2001) | Asia | 4 |
| J |  | 2007 | 2004 (2003-2005) | South America | 94 |  | **J*** |  | 2003 | 2001 (2001-2002) | Asia  North America | 0 |
| K |  | 2008 | 2006 (2005-2006) | North America | 96 |  | **K** |  | 2003 | 2002 (2002-2002) | Europa  South America | 4 |
| L |  | 2008 | 2005 (2005-2006 | South America | 98 |  | **L*** |  | 2004 | 2001 (2001-2002) | Asia  Oceania  Europa  North America | 0 |
| M |  | 2008 | 2006 (2006-2006) | North America Asia | 15 |  | **M** |  | 2004 | 2002 (2002-2002) | Europa | 100 |
| N* |  | 2008 | 2006 (2005-2007) | Asia  Europe  South America  Africa | 1 |  | **N** |  | 2004 | 2002 (2002-2002) | Asia  North America | 99 |
| O |  | 2008 | 2006 (2005-2006) | South America  Oceania | 97 |  | **O** |  | 2004 | 2002 (2002-2002) | Europe  North America | 14 |
|  |  |  |  |  |  |  | **P** |  | 2004 | 2002 (2002-2003) | Europa | 0 |
|  |  |  |  |  |  |  | **Q** |  | 2005 | 2004 (2003-2004) | Asia | 79 |
|  |  |  |  |  |  |  | **R** |  | 2005 | 2003 (2003-2004) | North America  Oceania | 88 |
|  |  |  |  |  |  |  | **S** |  | 2006 | 2004 (2003-2004) | North America  South America | 26 |
|  |  |  |  |  |  |  | **T** |  | 2006 | 2004 (2004-2004) | North America  Asia | 0 |
|  |  |  |  |  |  |  | **U*** |  | 2006 | 2004 (2004-2004) | North America  South America | 89 |
|  |  |  |  |  |  |  | **V*** |  | 2007 | 2006 (2005-2006) | Asia  South America  Africa  North America | 8 |
|  |  |  |  |  |  |  | **W** |  | 2007 | 2006 (2005-2006) | Asia | 1 |

***** Viruses that were transmitted to other countries after they arrived in Chile

**†** Clade nodes support into which the Chilean sequences are grouped according to the node posterior probability value

**Supplementary table 3.** Circulation period of each H1 IAV genetic cluster (based on HA1 amino acid sequences) by geographic region.

| Continent |  | Years range of H1 IAV circulation | | | | | | |
| --- | --- | --- | --- | --- | --- | --- | --- | --- |
|  |  | H1-cluster 1 |  | H1-cluster 2 |  | H1-cluster 3 |  | H1-cluster 4 |
| Asia |  | 1990-2000 |  | 1994-2008 |  | 2004-2008 |  | 2007-2008 |
| Oceania |  | 1990-2000 |  | 1999-2007 |  | 2004-2008 |  | 2007-2008 |
| Europe |  | 1990-2001 |  | 1999-2008 |  | 2006-2008 |  | 2004-2008 |
| North America |  | 1990-2001 |  | 2000-2008 |  | 2004-2008 |  | 2006-2008 |
| Africa |  | 1995-1998 |  | 1997-2007 |  | 2005-2007 |  | 2006-2008 |
| South America |  | 1997-2001 |  | 1999-2008 |  | 2006-2007 |  | 2007-2008 |
| Chile |  | 1996-2000 |  | 2000-2008 |  | 2006 |  | 2008 |
| Global |  | **1990-2001** |  | **1994-2008** |  | **2004-2008** |  | **2004-2008** |

**Supplementary table 4** Circulation period of each H3 IAV genetic cluster (based on HA1 amino acid sequences) by geographic region.

| Continent |  | Years range of H3 IAV circulation | | | | |
| --- | --- | --- | --- | --- | --- | --- |
|  |  | H3-cluster 1 |  | H3-cluster 2 |  | H3-cluster 3 |
| Asia |  | 1990-2002 |  | 1996-2005 |  | 2002-2008 |
| Oceania |  | 1990-1997 |  | 1997-2003 |  | 2002-2008 |
| Europe |  | 1990-1998 |  | 1997-2004 |  | 2002-2008 |
| North America |  | 1990-1998 |  | 1997-2003 |  | 2002-2008 |
| Africa |  | 1994-1998 |  | 1998-2003 |  | 2002-2008 |
| South America |  | 1991-1997 |  | 1998-2005 |  | 2003-2008 |
| Chile |  | 1994-1996 |  | 2000-2003 |  | 2003-2007 |
| Global |  | **1990-2002** |  | **1996-2005** |  | **2002-2008** |
